# 1-(3,4-Dihy­droxy­phen­yl)hexan-1-one

**DOI:** 10.1107/S1600536810022555

**Published:** 2010-06-23

**Authors:** Xiao-Chun Peng, Wei-Jun Huang, Xi Wang, Dao-Hong Wu, Zhu-Ping Xiao

**Affiliations:** a College of Chemistry & Chemical Engineering, Jishou University, Jishou 416000, People’s Republic of China

## Abstract

In the title compound, C_12_H_16_O_3_, a fully extened hexyl carbon chain is attached to a benzene ring; the mean planes formed by the atoms in the benzene ring and the hexa­none are inclined at an angle 8.5 (2)° with respect to each other. In the crystal, inter­molecular O—H⋯O hydrogen bonds join the mol­ecules into an infinite sheet.

## Related literature

For the biological activity of alkylcatechols, see: Buu-hoï & Seailles (1955 ▶); Buu-hoï & Xuong (1961 ▶); Miller *et al.* (1938 ▶); Xiao, Fang *et al.* (2007 ▶); Xiao, Xue *et al.* (2007 ▶). For related structures, see: Cheng *et al.* (2009 ▶); Wang *et al.* (2009 ▶).
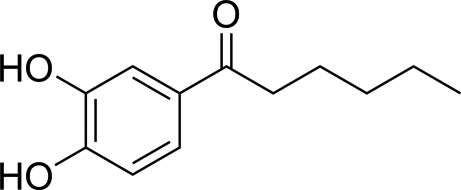


## Experimental

### 

#### Crystal data

C_12_H_16_O_3_
*M* *_r_* = 208.25
Triclinic, 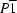

*a* = 7.170 (2) Å
*b* = 8.070 (3) Å
*c* = 10.634 (4) Å
α = 75.638 (17)°
β = 73.373 (19)°
γ = 88.323 (17)°
*V* = 570.6 (3) Å^3^
*Z* = 2
Mo *K*α radiation
μ = 0.09 mm^−1^
*T* = 296 K
0.30 × 0.24 × 0.20 mm

#### Data collection

Bruker SMART APEX CCD diffractometer
Absorption correction: multi-scan (*SADABS*; Sheldrick, 1996 ▶) *T*_min_ = 0.975, *T*_max_ = 0.983
3311 measured reflections
2321 independent reflections
1219 reflections with *I* > 2σ(*I*)
*R*_int_ = 0.016

#### Refinement

*R*[*F* ^2^ > 2σ(*F* ^2^)] = 0.065
*wR*(*F* ^2^) = 0.218
*S* = 1.00
2321 reflections
139 parameters
H-atom parameters constrained
Δρ_max_ = 0.27 e Å^−3^
Δρ_min_ = −0.30 e Å^−3^

### 

Data collection: *SMART* (Bruker, 2007 ▶); cell refinement: *SAINT* (Bruker, 2007 ▶); data reduction: *SAINT*; program(s) used to solve structure: *SHELXS97* (Sheldrick, 2008 ▶); program(s) used to refine structure: *SHELXL97* (Sheldrick, 2008 ▶); molecular graphics: *SHELXTL* (Sheldrick, 2008 ▶); software used to prepare material for publication: *SHELXL97*.

## Supplementary Material

Crystal structure: contains datablocks global, I. DOI: 10.1107/S1600536810022555/pv2291sup1.cif

Structure factors: contains datablocks I. DOI: 10.1107/S1600536810022555/pv2291Isup2.hkl

Additional supplementary materials:  crystallographic information; 3D view; checkCIF report

## Figures and Tables

**Table 1:** Hydrogen-bond geometry (Å, °)

*D*—H⋯*A*	*D*—H	H⋯*A*	*D*⋯*A*	*D*—H⋯*A*
O2—H2⋯O1	0.82	2.29	2.718 (2)	113
O2—H2⋯O1^i^	0.82	2.11	2.828 (3)	147
O1—H1*A*⋯O3^ii^	0.82	1.93	2.749 (2)	176

Symmetry codes: (i) 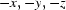; (ii) 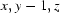.

**Fig. 1.**
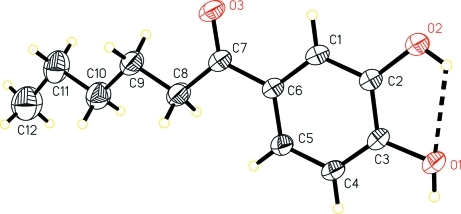
Molecular structure of the title compound, showing the atomic-numbering scheme. Displacement ellipsoids are drawn at the 30% probability level.

**Fig. 2.**
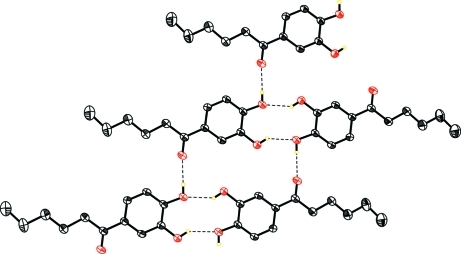
A two-dimensional sheet formed through intermolecular O—H···O hydrogen bonds.

## supplementary crystallographic information

### Comment

Acylcatechols are intermediates in the synthesis of alkycatechols which show significant bactericidal activity (Miller *et al.*, 1938; Xiao, Xue *et al.*, 2007; Xiao, Fang *et al.*, 2007) and possess considerable protective properties against lethal radiations (Buu-hoï & Seailles, 1955; Buu-hoï & Xuong, 1961). Consequently, we have synthesized a series of acylcatechols for bioactivity screening. In this paper, the crystal structure of the title compound has been presented. The crystal structures of compounds related to the title molecule have been reported (Cheng *et al.*, 2009; Wang *et al.*, 2009).

The bond lengths and angles in the title compound (Fig. 1) are unexceptional (Cheng *et al.*, 2009; Wang *et al.*, 2009). The hexyl carbon chain (C7—C12) attached to the phenyl ring is fully extened; the mean-planes formed by the atoms in the phenyl ring and hexanone are inclined at an angle 8.5 (2) ° with respect to each other. The two hydroxyl groups of catechol moiety are involved in an intramolecular hydrogen bond, O2—H2···O1 (Fig. 1). There are two O—H···O type intermolecular hydrogen bonds which stabilize the crystal structure and result in an infinite sheet (Fig. 2 and Tab. 1).

### Experimental

1-(3,4-Dihydroxyphenyl)hexan-1-one was prepared by treating hexanoic acid (1.27 g, 11 mmol) with catechol (1.11 g, 10 mmol) at 348 K for 4 h in boron trifluoride diethyl etherate (20 mL). After cooling, the contents were poured into 150 ml of ice-cold aqueous sodium acetate (10%) with stirring. Then, the mixture was extracted with EtOAc and dried over MgSO_4_. After removal of solvent, the crude product was crystalized from EtOAc-petroleum (2:3) to give colorless blocks of the title compound suitable for single-crystal structure determination.

### Refinement

All H atoms were placed in geometrically idealized positions and constrained to ride on their parent atoms with O—H = 0.82 Å and C—H = 0.93, 0.96 and 0.97 Å for the aromatic, CH_3_ and CH_2_ types H atoms, respectively. *U*_iso_ = 1.2*U*_eq_(parent atoms) were assigned for aromatic and CH_2_ type H-atoms and 1.5*U*_eq_(parent atoms) for OH and CH_3_ type H-atoms.

### Crystal data

**Table:**

C_12_H_16_O_3_	*Z* = 2
*M**_r_* = 208.25	*F*(000) = 224
Triclinic, *P*1	*D*_x_ = 1.212 Mg m^−^^3^
Hall symbol: -P 1	Mo *K*α radiation, λ = 0.71073 Å
*a* = 7.170 (2) Å	Cell parameters from 1104 reflections
*b* = 8.070 (3) Å	θ = 2.8–25.9°
*c* = 10.634 (4) Å	µ = 0.09 mm^−^^1^
α = 75.638 (17)°	*T* = 296 K
β = 73.373 (19)°	Block, colorless
γ = 88.323 (17)°	0.30 × 0.24 × 0.20 mm
*V* = 570.6 (3) Å^3^	

### Data collection

**Table:**

Bruker SMART APEX CCD diffractometer	2321 independent reflections
Radiation source: fine-focus sealed tube	1219 reflections with *I* > 2σ(*I*)
graphite	*R*_int_ = 0.016
φ and ω scans	θ_max_ = 26.5°, θ_min_ = 2.1°
Absorption correction: multi-scan (*SADABS*; Sheldrick, 1996)	*h* = −8→8
*T*_min_ = 0.975, *T*_max_ = 0.983	*k* = −10→10
3311 measured reflections	*l* = −13→13

### Refinement

**Table:**

Refinement on *F*^2^	Primary atom site location: structure-invariant direct methods
Least-squares matrix: full	Secondary atom site location: difference Fourier map
*R*[*F*^2^ > 2σ(*F*^2^)] = 0.065	Hydrogen site location: inferred from neighbouring sites
*wR*(*F*^2^) = 0.218	H-atom parameters constrained
*S* = 1.00	*w* = 1/[σ^2^(*F*_o_^2^) + (0.1157*P*)^2^ + 0.059*P*] where *P* = (*F*_o_^2^ + 2*F*_c_^2^)/3
2321 reflections	(Δ/σ)_max_ < 0.001
139 parameters	Δρ_max_ = 0.27 e Å^−^^3^
0 restraints	Δρ_min_ = −0.30 e Å^−^^3^

### Special details

**Table:**

Geometry. All e.s.d.'s (except the e.s.d. in the dihedral angle between two l.s. planes) are estimated using the full covariance matrix. The cell e.s.d.'s are taken into account individually in the estimation of e.s.d.'s in distances, angles and torsion angles; correlations between e.s.d.'s in cell parameters are only used when they are defined by crystal symmetry. An approximate (isotropic) treatment of cell e.s.d.'s is used for estimating e.s.d.'s involving l.s. planes.
Refinement. Refinement of *F*^2^ against ALL reflections. The weighted *R*-factor *wR* and goodness of fit *S* are based on *F*^2^, conventional *R*-factors *R* are based on *F*, with *F* set to zero for negative *F*^2^. The threshold expression of *F*^2^ > σ(*F*^2^) is used only for calculating *R*-factors(gt) *etc*. and is not relevant to the choice of reflections for refinement. *R*-factors based on *F*^2^ are statistically about twice as large as those based on *F*, and *R*- factors based on ALL data will be even larger.

### Fractional atomic coordinates and isotropic or equivalent isotropic displacement parameters (Å^2^)

**Table:**

	*x*	*y*	*z*	*U*_iso_*/*U*_eq_	
C1	0.2119 (4)	0.3836 (3)	0.1188 (3)	0.0558 (7)	
H1	0.1472	0.4851	0.1048	0.067*	
C2	0.1460 (4)	0.2413 (3)	0.0920 (3)	0.0565 (7)	
C3	0.2425 (4)	0.0894 (3)	0.1127 (3)	0.0540 (7)	
C4	0.4023 (4)	0.0824 (3)	0.1594 (3)	0.0627 (8)	
H4	0.4670	−0.0191	0.1730	0.075*	
C5	0.4682 (4)	0.2247 (3)	0.1864 (3)	0.0616 (8)	
H5	0.5768	0.2183	0.2183	0.074*	
C6	0.3743 (4)	0.3774 (3)	0.1665 (3)	0.0518 (7)	
C7	0.4394 (4)	0.5320 (3)	0.1971 (3)	0.0562 (7)	
C8	0.6048 (4)	0.5200 (3)	0.2579 (3)	0.0661 (8)	
H8A	0.7211	0.4946	0.1932	0.079*	
H8B	0.5762	0.4244	0.3376	0.079*	
C9	0.6490 (5)	0.6798 (4)	0.2985 (4)	0.0746 (9)	
H9A	0.6914	0.7730	0.2176	0.090*	
H9B	0.5302	0.7121	0.3565	0.090*	
C10	0.8016 (5)	0.6562 (4)	0.3708 (4)	0.0818 (10)	
H10A	0.9216	0.6285	0.3109	0.098*	
H10B	0.7617	0.5589	0.4489	0.098*	
C11	0.8435 (6)	0.8093 (5)	0.4187 (4)	0.1014 (12)	
H11A	0.7271	0.8344	0.4833	0.122*	
H11B	0.8805	0.9087	0.3422	0.122*	
C12	1.0097 (8)	0.7713 (6)	0.4859 (5)	0.1353 (17)	
H12A	0.9851	0.6605	0.5486	0.203*	
H12B	1.0161	0.8568	0.5333	0.203*	
H12C	1.1311	0.7729	0.4174	0.203*	
O1	0.1692 (3)	−0.0474 (2)	0.0840 (2)	0.0678 (6)	
H1A	0.2286	−0.1328	0.1064	0.102*	
O2	−0.0156 (3)	0.2520 (2)	0.0467 (3)	0.0810 (7)	
H2	−0.0279	0.1659	0.0214	0.121*	
O3	0.3591 (3)	0.6667 (2)	0.1739 (2)	0.0714 (7)	

### Atomic displacement parameters (Å^2^)

**Table:**

	*U*^11^	*U*^22^	*U*^33^	*U*^12^	*U*^13^	*U*^23^
C1	0.0566 (15)	0.0368 (13)	0.0788 (19)	0.0104 (11)	−0.0238 (14)	−0.0190 (12)
C2	0.0543 (15)	0.0413 (13)	0.081 (2)	0.0079 (11)	−0.0261 (14)	−0.0214 (13)
C3	0.0582 (16)	0.0348 (12)	0.0747 (19)	0.0041 (11)	−0.0228 (14)	−0.0200 (12)
C4	0.0632 (17)	0.0372 (13)	0.100 (2)	0.0137 (12)	−0.0371 (16)	−0.0240 (14)
C5	0.0594 (16)	0.0436 (14)	0.094 (2)	0.0102 (12)	−0.0393 (16)	−0.0209 (14)
C6	0.0511 (14)	0.0376 (13)	0.0712 (18)	0.0060 (11)	−0.0207 (13)	−0.0186 (12)
C7	0.0621 (17)	0.0395 (14)	0.0705 (18)	0.0052 (12)	−0.0218 (14)	−0.0172 (12)
C8	0.0774 (19)	0.0475 (15)	0.085 (2)	0.0059 (13)	−0.0375 (17)	−0.0205 (14)
C9	0.083 (2)	0.0506 (16)	0.102 (2)	0.0032 (15)	−0.0437 (19)	−0.0220 (16)
C10	0.089 (2)	0.073 (2)	0.092 (2)	−0.0020 (18)	−0.034 (2)	−0.0281 (18)
C11	0.117 (3)	0.097 (3)	0.105 (3)	−0.017 (2)	−0.035 (2)	−0.047 (2)
C12	0.145 (4)	0.132 (4)	0.162 (4)	−0.001 (3)	−0.073 (4)	−0.064 (3)
O1	0.0716 (13)	0.0383 (9)	0.1094 (16)	0.0105 (9)	−0.0433 (12)	−0.0283 (10)
O2	0.0760 (13)	0.0531 (11)	0.147 (2)	0.0214 (10)	−0.0673 (14)	−0.0462 (12)
O3	0.0833 (14)	0.0400 (10)	0.1058 (16)	0.0155 (9)	−0.0440 (12)	−0.0271 (10)

### Geometric parameters (Å, °)

**Table:**

C1—C2	1.376 (3)	C8—H8B	0.9700
C1—C6	1.392 (4)	C9—C10	1.491 (4)
C1—H1	0.9300	C9—H9A	0.9700
C2—O2	1.369 (3)	C9—H9B	0.9700
C2—C3	1.391 (3)	C10—C11	1.515 (4)
C3—C4	1.368 (3)	C10—H10A	0.9700
C3—O1	1.371 (3)	C10—H10B	0.9700
C4—C5	1.377 (4)	C11—C12	1.543 (6)
C4—H4	0.9300	C11—H11A	0.9700
C5—C6	1.385 (3)	C11—H11B	0.9700
C5—H5	0.9300	C12—H12A	0.9600
C6—C7	1.484 (3)	C12—H12B	0.9600
C7—O3	1.220 (3)	C12—H12C	0.9600
C7—C8	1.495 (4)	O1—H1A	0.8200
C8—C9	1.526 (4)	O2—H2	0.8200
C8—H8A	0.9700		
			
C2—C1—C6	120.8 (2)	C10—C9—C8	113.4 (3)
C2—C1—H1	119.6	C10—C9—H9A	108.9
C6—C1—H1	119.6	C8—C9—H9A	108.9
O2—C2—C1	119.0 (2)	C10—C9—H9B	108.9
O2—C2—C3	121.2 (2)	C8—C9—H9B	108.9
C1—C2—C3	119.7 (2)	H9A—C9—H9B	107.7
C4—C3—O1	123.5 (2)	C9—C10—C11	115.1 (3)
C4—C3—C2	119.8 (2)	C9—C10—H10A	108.5
O1—C3—C2	116.7 (2)	C11—C10—H10A	108.5
C3—C4—C5	120.5 (2)	C9—C10—H10B	108.5
C3—C4—H4	119.8	C11—C10—H10B	108.5
C5—C4—H4	119.8	H10A—C10—H10B	107.5
C4—C5—C6	120.7 (2)	C10—C11—C12	110.0 (3)
C4—C5—H5	119.7	C10—C11—H11A	109.7
C6—C5—H5	119.7	C12—C11—H11A	109.7
C5—C6—C1	118.5 (2)	C10—C11—H11B	109.7
C5—C6—C7	122.0 (2)	C12—C11—H11B	109.7
C1—C6—C7	119.5 (2)	H11A—C11—H11B	108.2
O3—C7—C6	120.8 (2)	C11—C12—H12A	109.5
O3—C7—C8	120.3 (2)	C11—C12—H12B	109.5
C6—C7—C8	119.0 (2)	H12A—C12—H12B	109.5
C7—C8—C9	115.3 (2)	C11—C12—H12C	109.5
C7—C8—H8A	108.4	H12A—C12—H12C	109.5
C9—C8—H8A	108.4	H12B—C12—H12C	109.5
C7—C8—H8B	108.4	C3—O1—H1A	109.5
C9—C8—H8B	108.4	C2—O2—H2	109.5
H8A—C8—H8B	107.5		

### Hydrogen-bond geometry (Å, °)

**Table:**

*D*—H···*A*	*D*—H	H···*A*	*D*···*A*	*D*—H···*A*
O2—H2···O1	0.82	2.29	2.718 (2)	113
O2—H2···O1^i^	0.82	2.11	2.828 (3)	147
O1—H1A···O3^ii^	0.82	1.93	2.749 (2)	176

Symmetry codes: (i) −*x*, −*y*, −*z*; (ii) *x*, *y*−1, *z*.
